# An association study in the Taiwan Biobank elicits the GABAA receptor genes *GABRB3*, *GABRA5*, and *GABRG3* as candidate loci for sleep duration in the Taiwanese population

**DOI:** 10.1186/s12920-021-01083-x

**Published:** 2021-09-16

**Authors:** Sheue-Jane Hou, Shih-Jen Tsai, Po-Hsiu Kuo, Wan-Yu Lin, Yu-Li Liu, Albert C. Yang, Eugene Lin, Tsuo-Hung Lan

**Affiliations:** 1 Institute of Clinical Medicine, National Yang Ming Chiao Tung University, Taipei, Taiwan; 2 Department of Psychiatry, Cheng Hsin General Hospital, Taipei, Taiwan; 3 Department of Psychiatry, Taipei Veterans General Hospital, Taipei, Taiwan; 4 Division of Psychiatry, National Yang Ming Chiao Tung University, Taipei, Taiwan; 5 Department of Public Health, Institute of Epidemiology and Preventive Medicine, National Taiwan University, Taipei, Taiwan; 6 Department of Psychiatry, National Taiwan University Hospital, Taipei, Taiwan; 7 Center for Neuropsychiatric Research, National Health Research Institutes, Miaoli County, Taiwan; 8 Division of Interdisciplinary Medicine and Biotechnology, Beth Israel Deaconess Medical Center, Harvard Medical School, Boston, MA 02215 USA; 9 Institute of Brain Science, National Yang Ming Chiao Tung University, Taipei, Taiwan; 10 Department of Biostatistics, University of Washington, 3980 15th Avenue NE, Box 351617, Seattle, WA 98195 USA; 11 Department of Electrical and Computer Engineering, University of Washington, Seattle, WA 98195 USA; 12 Graduate Institute of Biomedical Sciences, China Medical University, Taichung, Taiwan; 13 Tsaotun Psychiatric Center, Ministry of Health and Welfare, Nantou, Taiwan

**Keywords:** Biobank, GABAA receptor gene, Diverse populations, *GABRB3*-*GABRA5*-*GABRG3* gene cluster, Gene–gene interaction, Gene–environment interaction, Sleep duration

## Abstract

**Background:**

Gamma-aminobutyric acid type A (GABAA) receptors mainly mediate the effects of gamma-aminobutyric acid, which is the primary inhibitory neurotransmitter in the central nervous system. Abundant evidence suggests that GABAA receptors play a key role in sleep-regulating processes. No genetic association study has explored the relationships between GABAA receptor genes and sleep duration, sleep quality, and sleep timing in humans.

**Methods:**

We determined the association between single-nucleotide polymorphisms (SNPs) in the GABAA receptor genes *GABRA1*, *GABRA2*, *GABRB3*, *GABRA5*, and *GABRG3* and sleep duration, sleep quality, and sleep timing in the Taiwan Biobank with a sample of 10,127 Taiwanese subjects. There were 10,142 subjects in the original study cohort. We excluded 15 subjects with a medication history of sedative-hypnotics.

**Results:**

Our data revealed an association of the *GABRB3-GABRA5-GABRG3* gene cluster with sleep duration, which has not been previously identified: rs79333046 (beta = − 0.07; *P* = 1.21 × 10^–3^) in *GABRB3*, rs189790076 (beta = 0.92; *P* = 1.04 × 10^–3^) in *GABRA5*, and rs147619342 (beta = − 0.72; *P* = 3.97 × 10^–3^) in *GABRG3*. The association between rs189790076 in *GABRA5* and sleep duration remained significant after Bonferroni correction. A variant (rs12438141) in *GABRB3* was also found to act as a potential expression quantitative trait locus. Additionally, we discovered interactions between variants in the *GABRB3-GABRA5-GABRG3* gene cluster and lifestyle factors, such as tea and coffee consumption, smoking, and physical activity, that influenced sleep duration, although some interactions became nonsignificant after Bonferroni correction. We also found interactions among *GABRB3*, *GABRA5*, and *GABRG3* that affected sleep duration. Furthermore, we identified an association of rs7165524 (beta = − 0.06; *P* = 2.20 × 10^–3^) in *GABRA5* with sleep quality and an association of rs79465949 (beta = − 0.12; *P* = 3.95 × 10^–3^) in *GABRB3* with sleep timing, although these associations became nonsignificant after Bonferroni correction. However, we detected no evidence of an association of individual SNPs in *GABRA1* and *GABRA2*.

**Conclusions:**

Our results indicate that rs189790076 in *GABRA5* and gene–gene interactions among *GABRB3*, *GABRA5*, and *GABRG3* may contribute to sleep duration in the Taiwanese population.

**Supplementary Information:**

The online version contains supplementary material available at 10.1186/s12920-021-01083-x.

## Background

Principally, gamma-aminobutyric acid type A (GABAA) receptors are recognized as a crucial factor in sleep-regulating processes in the central nervous system [1]. In drug design and discovery to date, many of the drugs prescribed for insomnia (i.e., sleeping pills or sedative-hypnotic drugs) exert effects on GABAA receptors in terms of the physiology and pharmacology of sleep [2–5]. Molecular biology studies have demonstrated two modes of neuronal transmission in the brain that are distributed evenly, namely inhibition mediated by gamma-aminobutyric acid (GABA) and excitation mediated by glutamate [6, 7]. Moreover, the effects of GABA, the principal inhibitory neurotransmitter, are primarily mediated by GABAA receptors in the central nervous system [8]. Increasing evidence suggests that GABAA receptors play a pivotal role in regulating sleep and wake cyclical patterns (i.e., circadian rhythms) in humans [9, 10]. Numerous studies have implied a link between sleep disorders (i.e., circadian rhythm misalignment; for example, short/too little [≤ 6 h per day] or long/too much sleep [> 9 h per day] [11]) and alterations in the function of GABAA receptors [9, 12, 13]. Sleep is crucial for improving quality of life, and sleep duration is characterized as the total amount of sleep during a 24-h daily physiological cycle in the biological system [14, 15]. Few genetic variants have been revealed to be associated with sleep duration despite heritability accounting for approximately 40% of sleep duration [16, 17].

GABAA receptors are coded by GABAA receptor genes such as gamma-aminobutyric acid type A receptor subunit alpha1 (*GABRA1*). Studies have suggested a connection between GABAA receptor genes and sleep-related behaviors [18–21]. For example, Tsai et al. [19] indicated a significant association between zolpidem-induced complex sleep behaviors and the A15G variant in the GABAA receptor gene *GABRA1* in the Taiwanese population. Choi et al. [18] also reported that the rs4263535 single-nucleotide polymorphism (SNP) in the GABAA receptor gene *GABRA1* contributes to sleep induction time during sedation induction using intravenous midazolam in the Korean population. In addition, Blednov et al. [21] suggested that *Gabra1* or *Gabra2* knockout mice (i.e., with the deletion or loss of the *Gabra1* or *Gabra2* gene) exhibited significantly reduced sleep time, which was induced by GABAA agonists. Moreover, a study implied that *Gabra1* knockout mice spent markedly less time in rapid eye movement sleep but markedly more time in non-rapid eye movement sleep [20].

The *GABRB3*-*GABRA5*-*GABRG3* gene cluster consists of the GABAA receptor genes *GABRB3*, *GABRA5*, and *GABRG3* located on chromosome 15q12, which encode subunits beta3, alpha5, and gamma3, respectively [22, 23]. The *GABRB3*-*GABRA5*-*GABRG3* gene cluster has been the focus of attention for many years, and studies have suggested that it is involved in various psychiatric and neurological diseases with comorbid sleep disorders, such as panic disorder, autism spectrum disorders, Angelman and Prader–Willi syndrome, and epilepsy [24–28].

Although several other genes are associated with sleep-regulation processes [29], to the best of our knowledge, no study has addressed the impact of GABAA receptor genes such as *GABRA1*, *GABRA2*, *GABRB3*, *GABRA5*, and *GABRG3* on sleep duration in humans. GABAA receptor genes have been speculated to be linked to sleep duration because the encoded products of these genes (i.e., GABAA receptors) play a central role in the sleep and wake timing of the circadian rhythm system in humans [2, 4, 7]. A study also reported that lifestyle factors such as physical activity are linked to health outcomes [30]. In addition, several studies [31–35] in the Taiwan Biobank have conducted interaction analyses on various traits, including gene–gene, gene–environment, gene–age, and gene–gender interactions. One study suggested that interactions between the *RORA* gene and lifestyle factors, including tea consumption, coffee consumption, physical activity, alcohol consumption, and smoking, may influence sleep duration [31]. Based on the aforementioned considerations, we conducted the first genetic association study on the relationships between GABAA receptor genes such as *GABRA1*, *GABRA2*, *GABRB3*, *GABRA5*, and *GABRG3* and sleep duration, sleep quality, and sleep timing in Taiwanese subjects from the Taiwan Biobank. Although there are other GABAA receptor genes, this pilot study focused on these five genes (i.e., *GABRA1*, *GABRA2*, *GABRB3*, *GABRA5*, and *GABRG3*) due to more studies on these genes based on previous literature [18–28]. Moreover, we performed interaction analyses on sleep duration as per previously described methods [31–35].

## Materials and methods

### Subjects

Taiwanese participants were recruited from the Taiwan Biobank for the present study [32–40]. The Taiwan Biobank contains specimens and their related data from the general Taiwanese population from multiple recruitment centers across Taiwan from 2012 to the present (ongoing) [32–40]. Focused on providing collaboration opportunities for researchers, the Taiwan Biobank is mainly funded by the Taiwanese government, and it facilitates public health-related research projects related to local common chronic diseases [36].

Participants were included if they met the following criteria: (1) able to perform activities of daily living and (2) self-reported Taiwanese ancestry [37]. Individuals with a history of cancer or nonresidents of Taiwan were excluded [37]. Our original study cohort comprised 10,142 Taiwanese subjects. We further excluded individuals with a medication history of sedative-hypnotics (n = 15). In total, there were 10,127 individuals in our final study cohort.

Self-reported data on “sleep duration”, “sleep quality” and “sleep timing” from the Taiwan Biobank questionnaire were used in this study [31]. Sleep duration was defined as the average number of hours slept in a 24-h period [31]. Classifications of “1” for very poor, “2” for poor, “3” for fair, “4” for good and “5” for very good were used as measures for average sleep quality [31]. For sleep timing, six subgroups were defined based on the individual’s usual sleep onset time: (1) 0–4 AM, (2) 4–8 AM, (3) 8–12 AM, (4) 12–16 PM, (5) 16–20 PM, and (6) 20–24 PM, which is a standard method in the Taiwan Biobank as previously described [31]. As part of the questionnaire, participants were also asked if they were current tea/alcohol/coffee drinkers, if they were current smokers, and if they had performed any recent physical activity [31]. Participants who were currently drinking coffee at least three times per week were classified as current coffee drinkers [31]. Similarly, participants who were currently drinking tea at least one time per day were classified as current tea drinkers [31]. Participants with an alcohol intake of over 150 mL per week for more than 6 months were classified as current alcohol drinkers [31]. Participants who had been smoking for more than 6 months were classified as current smokers [31]. Performing physical activity was defined as having exercised more than three times per week, for over 30 min each time [31]. Status of chronic disorders was defined as whether the participant had had the following chronic disorders: arthritis, asthma, diabetes, and/or heart disease.

The Institutional Review Board of the Taiwan Biobank granted ethical approval for the study before it was conducted (approval number: 201506095RINC). Each subject signed an approved informed consent form. Relevant regulations and guidelines were applied to all experiments performed.

### Genotyping

With the manufacturer’s instructions, DNA was isolated from blood samples via QIAmp DNA blood kit (Qiagen, Valencia, CA, USA). Agarose gel electrophoresis determined the quality of isolated genomic DNA, with evaluation of the quantity made using spectrophotometry [41]. We used the custom Taiwan Biobank chips for SNP genotyping conducted on the Axiom Genome-Wide Arrray Plate System (Affymetrix, Santa Clara, CA, USA). SNPs on the Axiom Genome-Wide CHB 1 Array (Affymetrix) and the Human Exome BeadChip (Illumina, Inc., San Diego, CA, USA) were used to create custom Taiwan Biobank chips in order to efficiently extract maximal genetic information from Taiwanese samples [37]. Both the custom Taiwan Biobank chip and the Axiom Genome-Wide CHB 1 Array were used to genotype 70 unrelated Taiwanese individuals in order to accurately examine the performance of the chip previously mentioned. A high average concordance rate of 99.55% was achieved as a result for the SNPs in the 70 individuals [37].

In the present study, we searched for GABAA receptor-associated variants by referring to the complete list of GABAA receptor genes (such as *GABRA1*, *GABRA2*, *GABRB3*, *GABRA5*, and *GABRG3*) available in the custom Taiwan Biobank chips. Furthermore, the following quality control criteria were adopted for excluding SNPs from subsequent analysis [32, 42]: failure to achieve Hardy–Weinberg equilibrium (with a *P* value less than 0.05), minor allele frequency (MAF) < 1%, or a genotyping call rate less than 95%. After exclusion according to those criteria, we established independent SNPs that are not in linkage disequilibrium to each other using the LDlink tool [43] with an Asian reference panel. Finally, we obtained two SNP panels. The first SNP panel covered 43 SNPs from the following three GABAA receptor genes: *GABRB3*, *GABRA5*, and *GABRG3*. The second SNP panel covered five SNPs from the following two GABAA receptor genes: *GABRA1* and *GABRA2*.

### Statistical analysis

Chi-squared test was used to evaluate categorical data. Differences in the means of two continuous variables were compared by conducting the Student’s *t* test. Furthermore, we investigated the association of the investigated SNP with sleep duration, sleep quality, and sleep timing using linear regression analysis, adjusting for age, gender, physical activity, coffee consumption, tea consumption, smoking, alcohol consumption, body mass index (BMI), and status of chronic disorders as previously described [34, 44]. Sleep quality and sleep timing, captured as categorical variables, were analyzed as continuous variables in the genetic association analysis. We performed dominant, recessive, and genotypic genetic association tests using PLINK [42]. The dominant model was calculated using the major allele homozygous genotypes as references (i.e., major allele homozygous carriers vs. heterozygous and minor allele homozygous carriers grouped together). The recessive model was calculated using the heterozygous and major allele homozygous genotypes as references (i.e., heterozygous and major allele homozygous carriers grouped together vs. minor allele homozygous carriers). A chi-squared goodness-of-fit test with 1 degree of freedom (i.e. the number of genotypes minus the number of alleles) was used to assess the genotype frequencies for Hardy–Weinberg equilibrium [31]. Multiple testing was adjusted with Bonferroni correction [31]. For example, we divided 0.05 by the number of independent SNPs (i.e., 43) in the first SNP panel to obtain the significant *P* value (*P* < 0.05/43 = 1.16 × 10^–3^). Mean ± standard deviation were shown as a presentation of data [31].

In addition, we used the most significant SNP (in the dominant, recessive, or genotypic model) in a specific GABAA receptor gene of the *GABRB3*-*GABRA5*-*GABRG3* gene cluster (such as *GABRB3*, *GABRA5*, and *GABRG3*) to perform interaction analyses using R, which was a similar approach to that used in a recent study [31]. To investigate gene–gene and gene–environment interactions, we used multiple linear regression models and provided covariates such as age, gender, physical activity, coffee consumption, tea consumption, smoking, alcohol consumption, BMI, and status of chronic disorders in our interaction analyses based on previous literature [31]. The individuals were also stratified into five age groups by decade of life (30 s, 40 s, 50 s, 60 s, and 70 s), as previously described [31]. To investigate gene–age (using age groups) and gene–gender interactions, we used multiple linear regression models and provided covariates such as gender/age, physical activity, coffee consumption, tea consumption, smoking, alcohol consumption, BMI, and status of chronic disorders in our interaction analyses as previously described [31]. Moreover, we performed a stratified analysis for significant interactions. We also implemented interaction plots [45] to clarify whether interactions were significant in gene–environment interactions.

HaploReg (http://compbio.mit.edu/HaploReg) [46] was used to examine potential functional mechanisms, which means to estimate an SNP as an expression quantitative trait locus (eQTL).

## Results

### Taiwan Biobank sample

Table 1 presents the demographic characteristics of the study cohort, which comprised 10,127 individuals. The mean and the standard deviation values of sleep duration for overall, male, and female subjects were 6.6 ± 1.1, 6.7 ± 1.1, and 6.4 ± 1.2 h, respectively. In addition, the mean and standard deviation values of age for overall, male, and female subjects were 56.5 ± 9.4, 58.0 ± 9.8, and 56.1 ± 9.2 years, respectively.

**Table 1:** Demographic characteristics of the study subjects

Characteristic	Overall	Male	Female
No. of subjects, n	10,127	2,327	7,800
Mean age ± SD, years	56.5 ± 9.4	58.0 ± 9.8	56.1 ± 9.2
Sleep duration ± SD, hours per day	6.6 ± 1.1	6.7 ± 1.1	6.4 ± 1.2
Sleep quality, n (%)			
“1” for very poor	1: 271 (2.7)	1: 37 (1.6)	1: 234 (3.0)
“2” for poor	2: 1,927 (19.0)	2: 355 (15.3)	2: 1,572 (20.2)
“3” for fair	3: 4,374 (43.2)	3: 950 (40.8)	3: 3,424 (43.9)
“4” for good	4: 2,140 (21.1)	4: 571 (24.5)	4: 1,569 (20.1)
“5” for very good	5: 1,412 (13.8)	5: 414 (17.8)	5: 998 (12.8)
Sleep timing, n (%)			
(1) 0–4 AM	(1): 2,071 (20.5)	(1): 440 (18.9)	(1): 1,631 (20.9)
(2) 4–8 AM	(2): 23 (0.2)	(2): 11 (0.5)	(2): 12 (0.2)
(3) 8–12 AM	(3): 20 (0.2)	(3): 11 (0.5)	(3): 9 (0.1)
(4) 12–16 PM	(4): 7 (0.1)	(4): 0 (0.0)	(4): 7 (0.1)
(5) 16–20 PM	(5): 27 (0.3)	(5): 9 (0.4)	(5): 18 (0.2)
(6) 20–24 PM	(6): 7,941 (78.4)	(6): 1,830 (78.6)	(6): 6,111 (78.3)

Data are presented as mean ± standard deviation

Sleep quality is classified as “1” for very poor, “2” for poor, “3” for fair, “4” for good, and “5” for very good to evaluate the quality of usual sleep

Sleep timing is divided into six subgroups: (1) 0–4 AM, (2) 4–8 AM, (3) 8–12 AM, (4) 12–16 PM, (5) 16–20 PM, and (6) 20–24 PM based on usual sleep onset time

*SD* standard deviation

### Novel variants for sleep duration in the *GABRB3-GABRA5-GABRG3* gene cluster identified in the Taiwanese population

Among the 43 SNPs assessed in this study, there were six SNPs in the *GABRB3* gene, three SNPs in the *GABRA5* gene, and four SNPs in the *GABRG3* gene, which provided evidence of an association (*P* < 0.05) with sleep duration (Table 2).

**Table 2:** Linear regression models of associations between sleep duration and the GABAA receptor genes *GABRB3*, *GABRA5*, and *GABRG3*

Gene	Chr	SNP	A1	A2	Region	MAF	Dominant model			Recessive model			Genotypic model		
							Beta	SE	P	Beta	SE	P	Beta	SE	P
*GABRB3*	15	rs150101078	A	G	Intron	0.040	0.13	0.04	**2.61 × 10**^**−3**^	0.01	0.28	0.985	0.01	0.14	0.956
		rs12438141	T	C	Missense	0.059	0.07	0.04	**0.034**	− 0.02	0.19	0.927	0.00	0.10	0.962
		rs79565260	A	G	Intron	0.178	0.03	0.02	0.230	0.06	0.06	0.365	0.03	0.03	0.310
		rs149646336	T	C	Intron	0.027	0.00	0.05	0.939	0.15	0.43	0.728	0.07	0.21	0.728
		rs74591460	C	A	Intron	0.099	− 0.01	0.03	0.861	0.22	0.11	**0.040**	0.11	0.05	**0.043**
		rs79465949	T	C	Intron	0.018	0.01	0.06	0.839	0.65	0.56	0.249	0.32	0.28	0.249
		rs74004636	T	G	Intron	0.057	− 0.01	0.04	0.850	− 0.44	0.20	**0.024**	− 0.22	0.10	**0.024**
		rs79333046	A	G	Intron	0.213	− 0.07	0.02	**1.21 × 10**^**−3**^	− 0.07	0.06	0.207	− 0.05	0.03	0.088
		rs76635931	G	T	Intron	0.014	0.09	0.07	0.187	− 1.08	1.13	0.335	NA	NA	NA
		rs111863451	G	T	Intron	0.027	− 0.02	0.05	0.685	0.80	0.40	**0.045**	0.40	0.20	**0.046**
		rs145333058	T	C	Intron	0.015	0.02	0.07	0.716	− 0.54	0.80	0.495	− 0.27	0.40	0.495
		rs146937293	C	T	Intron	0.043	0.01	0.04	0.770	0.09	0.23	0.709	0.04	0.12	0.707
		rs77328500	G	A	Intron	0.077	− 0.02	0.03	0.442	0.03	0.15	0.860	0.01	0.08	0.879
		rs74653915	C	T	Intron	0.016	− 0.11	0.06	0.103	0.02	0.80	0.980	0.01	0.40	0.984
*GABRA5*	15	rs78575803	A	G	Intron	0.101	0.02	0.03	0.498	0.11	0.10	0.297	0.06	0.05	0.287
		rs7165524	T	C	Intron	0.270	− 0.02	0.02	0.311	− 0.02	0.04	0.721	− 0.01	0.02	0.573
		rs80063633	A	C	Intron	0.151	0.00	0.02	0.949	0.15	0.07	**0.045**	0.07	0.04	**0.049**
		rs140148185	A	G	Intron	0.090	− 0.01	0.03	0.809	0.39	0.13	**2.73 × 10**^**−3**^	0.19	0.06	**3.05 × 10**^**−3**^
		rs146013014	G	A	Intron	0.026	0.07	0.05	0.182	0.55	0.40	0.163	0.28	0.20	0.161
		rs189790076	C	G	Intron	0.016	0.06	0.06	0.316	1.84	0.56	**1.05 × 10**^**−3**^	0.92	0.28	**1.04 × 10**^**−3**^
		rs140319999	T	C	Intron	0.018	0.10	0.06	0.103	− 0.17	0.80	0.827	− 0.09	0.40	0.831
		rs34560927	T	C	Intron	0.372	0.00	0.02	0.988	0.06	0.03	0.091	0.02	0.02	0.174
*GABRG3*	15	rs34984550	G	C	Intron	0.024	0.00	0.05	0.941	− 0.18	0.56	0.752	− 0.09	0.28	0.752
		rs140670	A	T	Intron	0.293	0.01	0.02	0.664	− 0.03	0.04	0.455	− 0.01	0.02	0.586
		rs147619342	T	C	Intron	0.017	− 0.08	0.06	0.208	− 1.45	0.50	**4.02 × 10**^**−3**^	− 0.72	0.25	**3.97 × 10**^**−3**^
		rs12903002	T	C	Intron	0.088	0.02	0.03	0.490	0.01	0.13	0.921	0.01	0.07	0.901
		rs1017364	G	A	Intron	0.266	0.03	0.02	0.217	− 0.01	0.04	0.813	0.00	0.02	0.946
		rs150722146	A	G	Intron	0.026	− 0.05	0.05	0.282	− 0.29	0.56	0.604	− 0.15	0.28	0.601
		rs138047237	A	G	Intron	0.013	0.06	0.07	0.384	1.30	0.80	0.103	0.65	0.40	0.103
		rs734254	A	G	Intron	0.034	− 0.11	0.05	**0.018**	− 0.33	0.34	0.325	− 0.17	0.17	0.316
		rs12593313	G	C	Intron	0.022	0.00	0.06	0.989	− 0.01	0.43	0.990	0.00	0.21	0.990
		rs140853604	T	C	Intron	0.031	− 0.01	0.05	0.789	0.32	0.37	0.387	0.16	0.19	0.389
		rs7173587	T	C	Intron	0.157	− 0.06	0.02	**0.023**	0.08	0.07	0.288	0.03	0.04	0.419
		rs143682098	G	A	Intron	0.053	− 0.02	0.04	0.679	− 0.12	0.25	0.645	− 0.06	0.13	0.641
		rs17739682	G	A	Intron	0.072	0.02	0.03	0.620	0.12	0.17	0.489	0.06	0.08	0.483
		rs144369966	C	T	Intron	0.018	0.06	0.06	0.316	− 0.29	0.65	0.653	− 0.14	0.32	0.656
		rs67338000	A	G	Intron	0.449	− 0.04	0.02	0.068	0.00	0.03	0.859	− 0.01	0.02	0.401
		rs8028000	C	T	Intron	0.014	− 0.14	0.07	**0.041**	− 1.64	0.80	**0.039**	− 0.82	0.40	**0.039**
		rs61507711	T	C	Intron	0.097	0.02	0.03	0.582	0.10	0.12	0.384	0.05	0.06	0.376
		rs9302372	A	G	Intron	0.042	0.02	0.04	0.670	− 0.08	0.23	0.740	− 0.04	0.12	0.745
		rs4555109	T	G	Intron	0.222	0.04	0.02	0.104	0.01	0.05	0.825	0.01	0.03	0.624
		rs149403614	A	G	Intron	0.035	0.00	0.04	0.994	0.05	0.40	0.894	0.03	0.20	0.894
		rs28399529	G	A	3′ UTR	0.022	0.00	0.06	0.965	− 0.25	0.65	0.705	− 0.12	0.33	0.705

Analysis was obtained after adjustment for covariates, including age, gender, physical activity, coffee consumption, tea consumption, smoking, alcohol consumption, body mass index (BMI), and status of chronic disorders

*P* values < 0.05 represent the significant values and are shown in bold

*A1* minor allele, *A2* major allele, *GABAA* gamma-aminobutyric acid type A, *Beta* Beta coefficients, *Chr* chromosome, *MAF* minor allele frequency, *NA* not available, *SE* standard error

As described in Table 2, we identified the most significant SNP in *GABRB3*, *GABRA5*, and *GABRG3* as follows: the rs189790076 SNP (beta = 0.92; *P* = 1.04 × 10^–3^) in the intron region of the *GABRA5* gene, the rs79333046 SNP (beta = − 0.07; *P* = 1.21 × 10^–3^) in the intron region of the *GABRB3* gene, and the rs147619342 SNP (beta = − 0.72; *P* = 3.97 × 10^–3^) in the intron region of the *GABRG3* gene. Moreover, rs189790076 in *GABRA5* remained significant after applying Bonferroni correction (*P* < 0.05/43 = 1.16 × 10^–3^).

Furthermore, rs140148185 (beta = 0.39; *P* = 2.73 × 10^–3^; intron region) in *GABRA5* and rs150101078 (beta = 0.13; *P* = 2.61 × 10^–3^; intron region) in *GABRB3* exhibited a borderline significant trend with sleep duration (Table 2). In addition, we identified a nominal association of sleep duration with rs80063633 in *GABRA5*, four SNPs (rs12438141, rs74591460, rs74004636, and rs111863451) in *GABRB3*, and three SNPs (rs734254, rs7173587, and rs8028000) in *GABRG3* (Table 2).

In addition, the rs12438141 SNP in the missense region of the *GABRB3* gene was shown to be a potential eQTL in various tissues such as nerve and adipose tissues; this was reported previously [47].

### Gene–environment interactions in the *GABRB3-GABRA5-GABRG3* gene cluster for sleep duration identified in the Taiwanese population

As described in Table 3, we identified significant interaction models between *GABRB3* rs79333046 and lifestyle factors, including physical activity (*P* = 2.08 × 10^–3^), coffee consumption (*P* = 3.26 × 10^–4^), tea consumption (*P* = 3.66 × 10^–3^), and smoking (*P* = 0.0345). The effects of these models concerning physical activity and coffee consumption remained significant after Bonferroni correction (*P* < 0.05/15 = 3.33 × 10^–3^). These analysis results indicated probable gene–environment interactions between *GABRB3* and lifestyle factors such as physical activity and coffee consumption, respectively, which affected sleep duration. Additional file 2: Table S1 and Additional file 1: Figure S1 present the stratified analysis results and interaction plots for these two significant interactions, respectively. However, the plots and the stratified analysis did not show similar trends. While there was no variation of sleep duration for the genotypes of *GABRB3* rs79333046 in terms of physical activity and coffee consumption in the interaction plots (Additional file 1: Figure S1 (A) and (B), respectively), there was no such a non-variable trend of the effect sizes in the stratified analysis for these two interactions (Additional file 2: Table S1).

**Table 3:** Gene–environment interaction models identified by multiple linear regression analysis

Interaction model	*P* value
*GABRB3* rs79333046, physical activity	2.08 × 10^–3^
*GABRB3* rs79333046, coffee consumption	3.26 × 10^–4^
*GABRB3* rs79333046, tea consumption	3.66 × 10^–3^
*GABRB3* rs79333046, smoking	0.0345
*GABRB3* rs79333046, alcohol consumption	NS
*GABRA5* rs189790076, physical activity	NS
*GABRA5* rs189790076, coffee consumption	3.42 × 10^–3^
*GABRA5* rs189790076, tea consumption	NS
*GABRA5* rs189790076, smoking	9.35 × 10^–3^
*GABRA5* rs189790076, alcohol consumption	0.0134
*GABRG3* rs147619342, physical activity	NS
*GABRG3* rs147619342, coffee consumption	0.0112
*GABRG3* rs147619342, tea consumption	4.59 × 10^–3^
*GABRG3* rs147619342, smoking	0.0423
*GABRG3* rs147619342, alcohol consumption	NS

Analysis was obtained after adjustment for covariates, including age, gender, physical activity, coffee consumption, tea consumption, smoking, alcohol consumption, body mass index (BMI), and status of chronic disorders

*NS* nonsignificant

In addition, we identified significant interaction models between *GABRA5* rs189790076 and lifestyle factors, including coffee consumption (*P* = 3.42 × 10^–3^), smoking (*P* = 9.35 × 10^–3^), and alcohol consumption (*P* = 0.0134; Table 3). However, the effects of these models concerning these lifestyle factors did not remain significant after Bonferroni correction (*P* < 0.05/15 = 3.33 × 10^–3^).

Moreover, we identified significant interaction models between *GABRG3* rs147619342 and lifestyle factors, including coffee consumption (*P* = 0.0112), tea consumption (*P* = 4.59 × 10^–3^), and smoking (*P* = 0.0423; Table 3). However, the effects of these models did not remain significant after Bonferroni correction (*P* < 0.05/15 = 3.33 × 10^–3^).

### Gene–gene interactions in the *GABRB3-GABRA5-GABRG3* gene cluster for sleep duration identified in the Taiwanese population

As described in Table 4, we identified significant interaction models involving *GABRB3* rs79333046 and *GABRA5* rs189790076 (*P* = 2.01 × 10^–3^), *GABRA5* rs189790076 and *GABRG3* rs147619342 (*P* = 1.05 × 10^–3^), and *GABRB3* rs79333046 and *GABRG3* rs147619342 (*P* = 4.68 × 10^–3^). The effects of these models concerning the *GABRB3*-*GABRA5*-*GABRG3* gene cluster remained significant after Bonferroni correction (*P* < 0.05/3 = 0.0167). These analysis results indicated probable gene–gene interactions among these three genes, which affected sleep duration. Additional file 3: Table S2 presents the stratified analysis results for these significant interactions.

**Table 4:** Gene–gene interaction models identified by multiple linear regression analysis

Interaction model	*P* value
*GABRB3* rs79333046, *GABRA5* rs189790076	2.01 × 10^–3^
*GABRA5* rs189790076, *GABRG3* rs147619342	1.05 × 10^–3^
*GABRB3* rs79333046, *GABRG3* rs147619342	4.68 × 10^–3^

Analysis was obtained after adjustment for covariates, including age, gender, physical activity, coffee consumption, tea consumption, smoking, alcohol consumption, body mass index (BMI), and status of chronic disorders

### Gene–gender and gene-age interactions in the *GABRB3-GABRA5-GABRG3* gene cluster for sleep duration identified in the Taiwanese population

We identified significant interaction models involving *GABRB3* rs79333046 and gender (*P* = 8.14 × 10^–4^), *GABRA5* rs189790076 and gender (*P* = 3.79 × 10^–4^), and *GABRG3* rs147619342 and gender (*P* = 1.41 × 10^–3^). In addition, there were significant interaction models involving *GABRB3* rs79333046 and age groups (*P* = 3.58 × 10^–10^) as well as *GABRA5* rs189790076 and age groups (*P* = 2.24 × 10^–10^). Additional file 4: Table S3 presents the stratified analysis results for these significant interactions.

### Novel variants for sleep quality and sleep timing in the *GABRB3-GABRA5-GABRG3* gene cluster identified in the Taiwanese population

The association of the GABA receptor genes *GABRB3*, *GABRA5*, and *GABRG3* on sleep quality and sleep timing was further investigated. Among the 43 SNPs investigated in the present study, we identified six SNPs in the *GABRB3-GABRA5-GABRG3* gene cluster to be associated (*P* < 0.05) with sleep quality (Additional file 5: Table S4). The most significant SNP was the rs7165524 SNP (beta = − 0.06; *P* = 2.20 × 10^–3^) in the intron region of the *GABRA5* gene.

In addition, we identified six SNPs in the *GABRB3-GABRA5-GABRG3* gene cluster to be associated (*P* < 0.05) with sleep timing (Additional file 6: Table S5). The most significant SNP was the rs79465949 SNP (beta = − 0.12; *P* = 3.95 × 10^–3^) in the intron region of the *GABRB3* gene.

### No link of sleep duration with *GABRA1* and *GABRA2* in the Taiwanese population

Finally, the association between sleep duration and GABA receptor genes such as *GABRA1* and *GABRA2* was explored. Among the five SNPs (including the two SNPs in *GABRA1* and three SNPs in *GABRA2*) examined in the present study, no evidence of an association (*P* < 0.05) with sleep duration was identified (Additional file 7: Table S6).

The association of the GABA receptor genes *GABRA1* and *GABRA2* with sleep quality and sleep timing was further investigated. Among the five SNPs examined in the present study, we identified two (rs76707584 and rs16851626) in the *GABRA2* gene to be associated (*P* < 0.05) with sleep quality (Additional file 8: Table S7). In addition, no evidence of an association of the five SNPs (*P* < 0.05) with sleep timing was identified (data not shown).

## Discussion

The majority of genetic association studies for sleep duration have been conducted in European populations; it has been suggested that genetic association studies in globally underrepresented and diverse populations, such as the Taiwanese population, have great potential to identify novel biomarkers for common traits such as sleep duration across the human genome [48]. We conducted the first genetic association study of sleep duration, sleep quality, and sleep timing using the GABAA receptor genes *GABRA1*, *GABRA2*, *GABRB3*, *GABRA5*, and *GABRG3* in the Taiwan Biobank.

In the present study, we found an association of sleep duration with SNPs in the *GABRB3-GABRA5-GABRG3* gene cluster on chromosome 15 that has not been previously reported, such as rs79333046 in *GABRB3*, rs189790076 in *GABRA5*, and rs147619342 in *GABRG3*. To the best of our knowledge, this is the first study to report a probable association between sleep duration and the GABAA receptor genes *GABRB3*, *GABRA5*, and *GABRG3*. *GABRB3*, *GABRA5*, and *GABRG3,* which are all located on chromosome 15q12 and encode the alpha5, beta3, and gamma3 subunits of GABAA receptors, respectively [49, 50]. Common in many neurological and psychiatric disorders in humans, GABAA receptors have been indicated to be involved in sleep disorders and sleep disturbances [2, 4, 7]. And these are encoded products of the three genes as a gene cluster namely the *GABRB3-GABRA5-GABRG3* gene cluster, a promising candidate locus for sleep duration. Furthermore, it has been reported that the *GABRB3*-*GABRA5*-*GABRG3* gene cluster may lead to increased susceptibility to a variety of neurological and psychiatric disorders with comorbid sleep disturbances, such as panic disorder, autism spectrum disorders, Angelman and Prader–Willi syndrome, and epilepsy [24–28]. For instance, Hodge et al. [25] reported that SNPs in the GABAA receptor genes *GABRB3* and *GABRA5* were associated with panic disorder in a Caucasian population. Notably, sleep-related behaviors such as sleep disturbance and sleep disorders are associated with patients with psychiatric diagnoses such as panic disorder [51].

We identified 13 SNPs in total in the *GABRB3-GABRA5-GABRG3* gene cluster to be associated with sleep duration (Table 2). We also found that the rs189790076 SNP in *GABRA5* maintained a significant association with sleep duration after Bonferroni correction. The probable effects of these SNPs in the *GABRB3-GABRA5-GABRG3* gene cluster on sleep duration remain to be investigated. In the present study, we employed HaploReg to investigate potential functional mechanisms for these SNPs. Remarkably, we found that the rs12438141 SNP in the missense region of the *GABRB3* gene served as a probable eQTL in nerve and adipose tissues [47] (see the Results section). A study reported that adipose tissue may play a key role in obstructive sleep apnea [52], a common sleep-related breathing disorder. Moreover, evidence of sensory nerve injury or dysfunction in obstructive sleep apnea was reported [53].

Based on the beta coefficient value for 79333046 in *GABRB3* (Table 2; beta = -0.07, dominant model), the carriers of the AA and AG genotypes had lower sleep duration than the carriers of the GG genotype. Based on the beta coefficient value for rs189790076 in *GABRA5* (Table 2; beta = 1.84, recessive model), the carriers of the CC genotype had higher sleep duration than the carriers of the CG and GG genotypes. Based on the beta coefficient value for rs147619342 in *GABRG3* (Table 2; beta = -1.45, recessive model), the carriers of the TT genotype had lower sleep duration than the carriers of the TC and CC genotypes.

In the present study, our data revealed that gene–environment interactions with the *GABRB3-GABRA5-GABRG3* gene cluster may be involved in the etiology of sleep duration. We found a significant association between the *GABRB3-GABRA5-GABRG3* gene cluster and lifestyle factors, including tea consumption, coffee consumption, smoking, and physical activity. This interaction might be demonstrated through epigenetic modifications. However, some associations concerning lifestyle factors with *GABRB3* rs79333046, *GABRA5* rs189790076, and *GABRG3* rs147619342 became nonsignificant after Bonferroni correction. There was also an inconsistent trend between the stratified analysis and the interaction plots for some significant associations concerning physical activity and coffee consumption with *GABRB3* rs79333046. In addition to revealing the statistical significance of gene–environment interactions, we investigated the potential biological role underlying the interactions. The functional consequences of the interactions of the *GABRB3-GABRA5-GABRG3* gene cluster with lifestyle factors in sleep duration remain to be elucidated. Similarly, Hou et al. [31] found a significant association between the circadian clock gene *RORA* and lifestyle factors, such as tea consumption, alcohol consumption, coffee consumption, smoking, and physical activity in affecting sleep duration in the Taiwanese population. A significant association between alcohol dependence and the GABAA receptor genes *GABRB3* and *GABRA5* was also revealed in a family-based association study in the Caucasian population [54]. Similarly, Dick et al. [55] observed that *GABRG3* was associated with alcohol dependence in another family-based association study. In addition, Lopez et al. [56] reported that an interaction of caffeine with GABAA receptors may result in alterations in the transport function of GABAA receptors, and caffeine is the well-known stimulant in tea and coffee. Moreover, it has been suggested that major noncommunicable diseases are associated with the lack of physical activity worldwide [30]. However, to the best of our knowledge, no study has investigated the relationship between the *GABRB3-GABRA5-GABRG3* gene cluster and lifestyle factors in affecting sleep duration. Based on previous findings [30, 31, 54–56], synergistic interactions between the *GABRB3-GABRA5-GABRG3* gene cluster and lifestyle factors on sleep duration may be a hallmark of GABAA receptors in sleep and wake cyclical mechanisms.

We further inferred the epistatic effects of the *GABRB3-GABRA5-GABRG3* gene cluster on sleep duration. To the best of our knowledge, our study is the first investigation of gene–gene interactions of the GABAA receptor genes *GABRB3*, *GABRA5*, and *GABRG3* with sleep duration. Similarly, in a family-based association study, Ma et al. [57] identified potential gene–gene interactions of GABAA receptor genes *GABRA4* and *GABRB1* in the etiology of autism in a population with European ancestry. By contrast, Ashley-Koch et al. [58] detected no significant gene–gene interactions in the *GABRB3-GABRA5-GABRG3* gene cluster that contributed to autism risk in a family-based association study in the Caucasian population. These findings suggest that the *GABRA5* gene is closely related to the *GABRB3* gene at the molecular level [22]. From a structural point of view, the *GABRB3* gene is adjacent to the *GABRA5* gene (approximately 100 kb apart) [59]. In a similar way, the *GABRA5* gene is next to the *GABRG3* gene (approximately 20 kb apart) [59]. In accordance with the aforementioned studies [22, 57–59], the present data indicate that potential allied reactions may exist among *GABRB3*, *GABRA5*, and *GABRG3* in sleep duration, and the mechanisms may synergistically integrate with each other.

Furthermore, we found gene–gender and gene–age interactions of the *GABRB3-GABRA5-GABRG3* gene cluster that influenced sleep duration. In accordance with our results, it has been indicated that sleep duration depends on both gender and age [31, 60]. Additionally, we found evidence of associations of the GABAA receptor genes *GABRB3*, *GABRA5*, and *GABRG3* with sleep quality and sleep timing, although these associations were not statistically significant after Bonferroni correction. To the best of our knowledge, no other studies have explored the relationships of these genes with sleep quality and sleep timing.

Finally, a link between sleep-related behaviors and the GABAA receptor genes *GABRA1* and *GABRA2* (or *Gabra1* and *Gabra2*) has been found in various human and animal studies [18–21]. However, we did not detect an association of *GABRA1* and *GABRA2* with sleep duration in the Taiwanese population. In addition, we found evidence of associations of the GABAA receptor gene *GABRA2* with sleep quality, although these associations were not statistically significant after Bonferroni correction.

This study has not only significant strengths but also some main weaknesses. A noteworthy strength is the considerable number of subjects from the Taiwan Biobank. By contrast, the major weakness is that the analysis was based on self-reported measures for sleep duration, timing, and quality. Furthermore, in our statistical analyses, adjustments were not made for an essential variable, namely shift work, due to a lack of such data.

## Conclusions

In summary, our study suggests that rs189790076 in *GABRA5* and gene–gene interactions among *GABRB3*, *GABRA5*, and *GABRG3* may play a key role in sleep duration in the Taiwan Biobank. We also identified an association between rs7165524 in *GABRA5* and sleep quality as well as an association between rs79465949 in *GABRB3* and sleep timing, although these associations did not remain significant after Bonferroni correction. This genetic association study of sleep duration, sleep quality, and sleep timing in the Taiwan Biobank implicates the need for genetic association studies in worldwide underrepresented populations, such as the Taiwanese population. Even though substantial genetic association studies on common traits, such as sleep duration, have been conducted mainly in populations with European ancestry, such studies should be conducted in worldwide underrepresented populations for discovering novel loci. In the present study, we employed the Taiwan Biobank, which is the largest Taiwanese genetic database to date, to explore the SNPs related to sleep duration, sleep quality, and sleep timing in GABAA receptor genes. In future work, we expect to achieve more new genetic discoveries for common and/or complex traits in the Taiwanese population when the Taiwan Biobank proceeds to increase in volume.

## Supplementary Information

**Additional file 1 Figure S1.** Interaction plots for significant interactions between *GABRB3* rs79333046 and lifestyle factors.

**Additional file 2 Table S1.** Stratified analysis results for interactions between *GABRB3* rs79333046 and lifestyle factors.

**Additional file 3 Table S2.** Stratified analysis results for gene–gene interactions.

**Additional file 4 Table S3.** Stratified analysis results for gene–gender and gene–age interactions.

**Additional file 5 Table S4.** Linear regression models of associations between sleep quality and six SNPs in GABAA receptor genes (e.g., *GABRB3*, *GABRA5*, and *GABRG3*) with evidence of an association (P < 0.05).

**Additional file 6 Table S5.** Linear regression models of associations between sleep timing and six SNPs in GABAA receptor genes (e.g., *GABRB3*, *GABRA5*, and *GABRG3*) with evidence of an association (P < 0.05).

**Additional file 7 Table S6.** Linear regression models of associations between sleep duration and five SNPs in GABAA receptor genes (e.g., *GABRA1* and *GABRA2*).

**Additional file 8.** Table S7. Linear regression models of associations between sleep quality and two SNPs in the GABAA receptor gene *GABRA2* with evidence of an association (P < 0.05).

## Data Availability

The data that support the findings of this study are available from the Taiwan Biobank. To apply for access to these third party data, please contact the Taiwan Biobank at biobank@gate.sinica.edu.tw.

## Abbreviations

eQTL: Expression quantitative trait locus
GABA: Gamma-aminobutyric acid
GABAA: Gamma-aminobutyric acid type A
*GABRA1*: Gamma-aminobutyric acid type A receptor subunit alpha1
MAF: Minor allele frequency
SNPs: Single-nucleotide polymorphisms
