# Supplementary material for: An association study in the Taiwan Biobank elicits the GABAA receptor genes GABRB3, GABRA5, and GABRG3 as candidate loci for sleep duration in the Taiwanese population
Source: BMC Med Genomics. 2021 Sep 16;14:223. doi: 10.1186/s12920-021-01083-x (PMC8447520; doi:10.1186/s12920-021-01083-x)
Supplement: Supplementary file 1 — Additional file 1 Figure S1. Interaction plots for significant interactions between GABRB3 rs79333046 and lifestyle factors. [file 12920_2021_1083_MOESM1_ESM.pdf]

(A) *GABRB3* rs79333046 and physical activity.

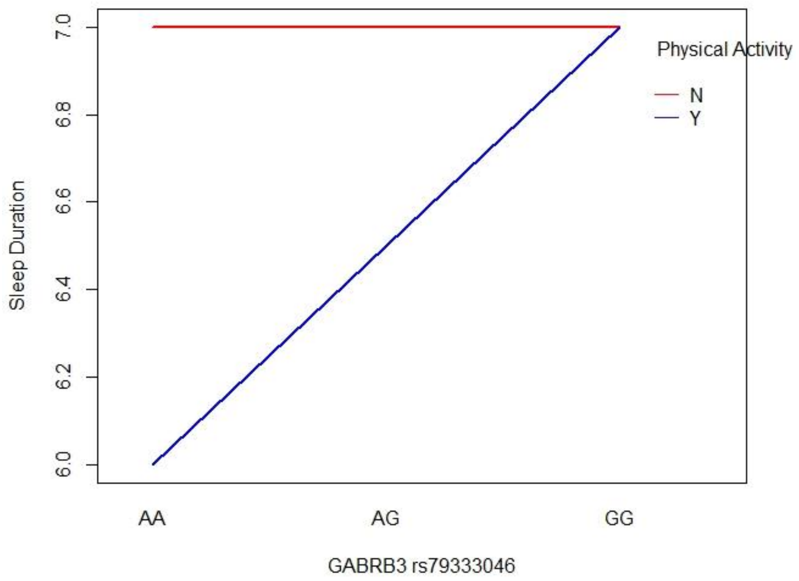

(B) *GABRB3* rs79333046 and coffee consumption.

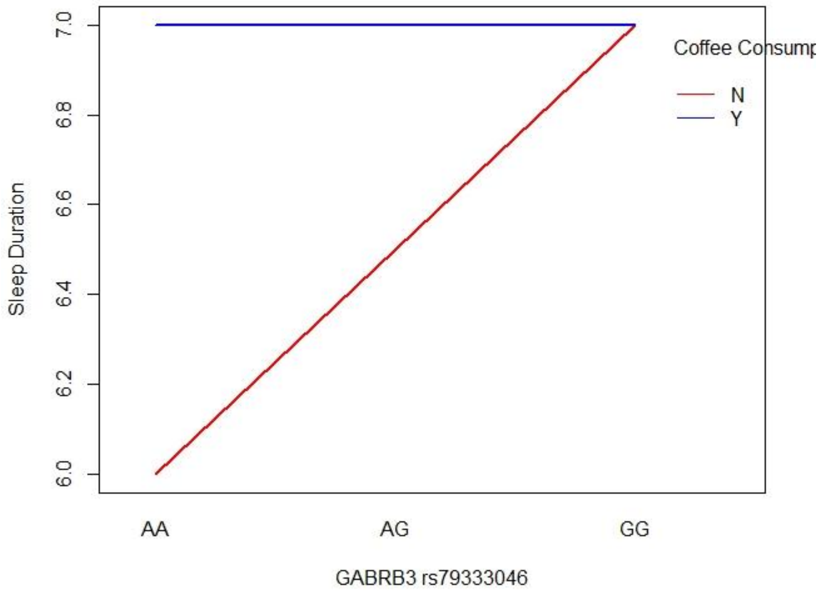

Figure S1. Interaction plots for significant interactions between *GABRB3* rs79333046 and lifestyle factors.
