# Supplementary material for: An association study in the Taiwan Biobank elicits the GABAA receptor genes GABRB3, GABRA5, and GABRG3 as candidate loci for sleep duration in the Taiwanese population
Source: BMC Med Genomics. 2021 Sep 16;14:223. doi: 10.1186/s12920-021-01083-x (PMC8447520; doi:10.1186/s12920-021-01083-x)
Supplement: Supplementary file 2 — Additional file 2 Table S1. Stratified analysis results for interactions between GABRB3 rs79333046 and lifestyle factors. [file 12920_2021_1083_MOESM2_ESM.pdf]

Table S1. Stratified analysis results for interactions between *GABRB3* rs79333046 and lifestyle factors.

Gene	Lifestyle factors	Beta	SE	P
GABRB3 rs79333046 (AA)	physical activity (N)	0.06	0.08	0.474
GABRB3 rs79333046 (AG)	physical activity (N)	-0.03	0.03	0.394
GABRB3 rs79333046 (GG)	physical activity (N)	0.03	0.03	0.312
GABRB3 rs79333046 (AA)	physical activity (Y)	-0.22	0.08	**5.90E-03**
GABRB3 rs79333046 (AG)	physical activity (Y)	-0.08	0.03	**0.017**
GABRB3 rs79333046 (GG)	physical activity (Y)	NA	NA	NA
GABRB3 rs79333046 (AA)	coffee consumption (N)	-0.27	0.07	**3.99E-04**
GABRB3 rs79333046 (AG)	coffee consumption (N)	-0.11	0.03	**9.73E-04**
GABRB3 rs79333046 (GG)	coffee consumption (N)	-0.04	0.03	0.178
GABRB3 rs79333046 (AA)	coffee consumption (Y)	0.08	0.08	0.361
GABRB3 rs79333046 (AG)	coffee consumption (Y)	-0.07	0.04	0.069
GABRB3 rs79333046 (GG)	coffee consumption (Y)	NA	NA	NA

Beta = beta coefficients, NA = not available, SE = standard error

P values <0.05 represent the significant values and are shown in bold.
