# Supplementary material for: An association study in the Taiwan Biobank elicits the GABAA receptor genes GABRB3, GABRA5, and GABRG3 as candidate loci for sleep duration in the Taiwanese population
Source: BMC Med Genomics. 2021 Sep 16;14:223. doi: 10.1186/s12920-021-01083-x (PMC8447520; doi:10.1186/s12920-021-01083-x)
Supplement: Supplementary file 3 — Additional file 3 Table S2. Stratified analysis results for gene–gene interactions. [file 12920_2021_1083_MOESM3_ESM.pdf]

Table S2. Stratified analysis results for gene–gene interactions.

Gene	Gene	Beta	SE	P
GABRB3 rs79333046 (AA)	*GABRA5* rs189790076 (CC)	NA	NA	NA
GABRB3 rs79333046 (AG)	*GABRA5* rs189790076 (CC)	0.82	1.12	0.466
GABRB3 rs79333046 (GG)	*GABRA5* rs189790076 (CC)	2.15	0.65	**9.45E-04**
GABRB3 rs79333046 (AA)	*GABRA5* rs189790076 (CG)	-0.42	0.50	0.403
GABRB3 rs79333046 (AG)	*GABRA5* rs189790076 (CG)	-0.04	0.12	0.715
GABRB3 rs79333046 (GG)	*GABRA5* rs189790076 (CG)	0.05	0.08	0.547
GABRB3 rs79333046 (AA)	*GABRA5* rs189790076 (GG)	-0.09	0.06	0.114
GABRB3 rs79333046 (AG)	*GABRA5* rs189790076 (GG)	-0.07	0.02	**4.90E-03**
GABRB3 rs79333046 (GG)	*GABRA5* rs189790076 (GG)	NA	NA	NA
GABRA5 rs189790076 (CC)	*GABRG3* rs147619342 (CC)	3.29	0.75	**1.29E-05**
GABRA5 rs189790076 (CG)	*GABRG3* rs147619342 (CC)	1.49	0.51	**3.34E-03**
GABRA5 rs189790076 (GG)	*GABRG3* rs147619342 (CC)	1.45	0.50	**3.90E-03**
GABRA5 rs189790076 (CC)	*GABRG3* rs147619342 (TC)	NA	NA	NA
GABRA5 rs189790076 (CG)	*GABRG3* rs147619342 (TC)	1.62	0.68	**0.017**
GABRA5 rs189790076 (GG)	*GABRG3* rs147619342 (TC)	1.39	0.51	**6.00E-03**
GABRA5 rs189790076 (CC)	*GABRG3* rs147619342 (TT)	NA	NA	NA
GABRA5 rs189790076 (CG)	*GABRG3* rs147619342 (TT)	NA	NA	NA
GABRA5 rs189790076 (GG)	*GABRG3* rs147619342 (TT)	NA	NA	NA
GABRB3 rs79333046 (AA)	*GABRG3* rs147619342 (CC)	1.40	0.56	**0.013**
GABRB3 rs79333046 (AG)	*GABRG3* rs147619342 (CC)	1.42	0.56	**0.012**
GABRB3 rs79333046 (GG)	*GABRG3* rs147619342 (CC)	1.49	0.56	**8.20E-03**
GABRB3 rs79333046 (AA)	*GABRG3* rs147619342 (TC)	1.07	0.64	0.095
GABRB3 rs79333046 (AG)	*GABRG3* rs147619342 (TC)	1.34	0.57	**0.019**
GABRB3 rs79333046 (GG)	*GABRG3* rs147619342 (TC)	1.46	0.57	**0.010**
GABRB3 rs79333046 (AA)	*GABRG3* rs147619342 (TT)	0.02	1.26	0.987
GABRB3 rs79333046 (AG)	*GABRG3* rs147619342 (TT)	NA	NA	NA
GABRB3 rs79333046 (GG)	*GABRG3* rs147619342 (TT)	NA	NA	NA

Beta = beta coefficients, NA = not available, SE = standard error

P values <0.05 represent the significant values and are shown in bold.
