# Supplementary material for: An association study in the Taiwan Biobank elicits the GABAA receptor genes GABRB3, GABRA5, and GABRG3 as candidate loci for sleep duration in the Taiwanese population
Source: BMC Med Genomics. 2021 Sep 16;14:223. doi: 10.1186/s12920-021-01083-x (PMC8447520; doi:10.1186/s12920-021-01083-x)
Supplement: Supplementary file 4 — Additional file 4 Table S3. Stratified analysis results for gene–gender and gene–age interactions. [file 12920_2021_1083_MOESM4_ESM.pdf]

Table S3. Stratified analysis results for gene–gender and gene–age interactions

Gene	Gender / Age Group	Beta	SE	P
GABRB3 rs79333046 (AA)	gender (F)	-0.17	0.07	**0.014**
GABRB3 rs79333046 (AG)	gender (F)	-0.14	0.04	**3.20E-04**
GABRB3 rs79333046 (GG)	gender (F)	-0.05	0.04	0.177
GABRB3 rs79333046 (AA)	gender (M)	-0.01	0.12	0.957
GABRB3 rs79333046 (AG)	gender (M)	0.00	0.05	0.940
GABRB3 rs79333046 (GG)	gender (M)	NA	NA	NA
GABRA5 rs189790076 (CC)	gender (F)	1.75	0.56	**1.83E-03**
GABRA5 rs189790076 (CG)	gender (F)	-0.01	0.08	0.880
GABRA5 rs189790076 (GG)	gender (F)	-0.09	0.03	**2.40E-03**
GABRA5 rs189790076 (CC)	gender (M)	NA	NA	NA
GABRA5 rs189790076 (CG)	gender (M)	-0.11	0.15	0.445
GABRA5 rs189790076 (GG)	gender (M)	NA	NA	NA
GABRG3 rs147619342 (CC)	gender (F)	2.21	1.13	0.050
GABRG3 rs147619342 (TC)	gender (F)	2.10	1.13	0.063
GABRG3 rs147619342 (TT)	gender (F)	0.96	1.26	0.447
GABRG3 rs147619342 (CC)	gender (M)	2.28	1.13	**0.043**
GABRG3 rs147619342 (TC)	gender (M)	2.37	1.13	**0.037**
GABRG3 rs147619342 (TT)	gender (M)	NA	NA	NA
GABRB3 rs79333046 (AA)	age group (30s)	0.16	0.21	0.436
GABRB3 rs79333046 (AG)	age group (30s)	0.11	0.10	0.291
GABRB3 rs79333046 (GG)	age group (30s)	0.22	0.09	**0.011**
GABRB3 rs79333046 (AA)	age group (40s)	0.21	0.16	0.189
GABRB3 rs79333046 (AG)	age group (40s)	0.09	0.08	0.281
GABRB3 rs79333046 (GG)	age group (40s)	0.12	0.08	0.107
GABRB3 rs79333046 (AA)	age group (50s)	-0.02	0.11	0.877
GABRB3 rs79333046 (AG)	age group (50s)	-0.07	0.07	0.369
GABRB3 rs79333046 (GG)	age group (50s)	0.02	0.07	0.739
GABRB3 rs79333046 (AA)	age group (60s)	-0.21	0.11	0.051
GABRB3 rs79333046 (AG)	age group (60s)	-0.12	0.07	0.096
GABRB3 rs79333046 (GG)	age group (60s)	-0.07	0.07	0.293
GABRB3 rs79333046 (AA)	age group (70s)	-0.72	0.25	**3.69E-03**
GABRB3 rs79333046 (AG)	age group (70s)	-0.19	0.11	0.074
GABRB3 rs79333046 (GG)	age group (70s)	NA	NA	NA
GABRA5 rs189790076 (CC)	age group (30s)	NA	NA	NA
GABRA5 rs189790076 (CG)	age group (30s)	0.68	0.26	**9.50E-03**
GABRA5 rs189790076 (GG)	age group (30s)	0.27	0.07	**1.18E-04**
GABRA5 rs189790076 (CC)	age group (40s)	1.01	1.13	0.368
GABRA5 rs189790076 (CG)	age group (40s)	0.37	0.17	**0.029**

Gene	Gender / Age Group	Beta	SE	P
GABRA5 rs189790076 (GG)	age group (40s)	0.21	0.06	**5.48E-04**
GABRA5 rs189790076 (CC)	age group (50s)	NA	NA	NA
GABRA5 rs189790076 (CG)	age group (50s)	0.12	0.12	0.305
GABRA5 rs189790076 (GG)	age group (50s)	0.09	0.06	0.097
GABRA5 rs189790076 (CC)	age group (60s)	1.55	0.80	0.052
GABRA5 rs189790076 (CG)	age group (60s)	-0.03	0.11	0.811
GABRA5 rs189790076 (GG)	age group (60s)	0.01	0.05	0.901
GABRA5 rs189790076 (CC)	age group (70s)	3.40	1.13	**2.55E-03**
GABRA5 rs189790076 (CG)	age group (70s)	-0.14	0.29	0.618
GABRA5 rs189790076 (GG)	age group (70s)	NA	NA	NA

Beta = beta coefficients, NA = not available, SE = standard error

P values <0.05 represent the significant values and are shown in bold.
