# Supplementary material for: An association study in the Taiwan Biobank elicits the GABAA receptor genes GABRB3, GABRA5, and GABRG3 as candidate loci for sleep duration in the Taiwanese population
Source: BMC Med Genomics. 2021 Sep 16;14:223. doi: 10.1186/s12920-021-01083-x (PMC8447520; doi:10.1186/s12920-021-01083-x)
Supplement: Supplementary file 5 — Additional file 5 Table S4. Linear regression models of associations between sleep quality and six SNPs in GABAA receptor genes (e.g., GABRB3, GABRA5, and GABRG3) with evidence of an association (P < 0.05). [file 12920_2021_1083_MOESM5_ESM.pdf]

Table S4. Linear regression models of associations between sleep quality and six SNPs in GABAA receptor genes (e.g., *GABRB3* , *GABRA5* , and *GABRG3*) with evidence of an association ($P < 0.05$).

							Dominant model			Recessive model			Genotypic model		
**Gene**	**Chr**	**SNP**	**A1**	**A2**	**Region**	**MAF**	**Beta**	**SE**	**P**	**Beta**	**SE**	**P**	**Beta**	**SE**	**P**
GABRB3	15	rs150101078	A	G	Intron	0.040	0.08	0.04	**0.032**	-0.15	0.25	0.544	-0.07	0.12	0.562
		rs74591460	C	A	Intron	0.099	0.04	0.03	0.155	0.22	0.09	**0.018**	0.11	0.05	**0.016**
GABRA5	15	rs78575803	A	G	Intron	0.101	0.01	0.03	0.820	0.23	0.09	**0.013**	0.11	0.05	**0.014**
		rs7165524	T	C	Intron	0.270	-0.06	0.02	**2.20E-03**	-0.07	0.04	0.076	-0.05	0.02	**0.020**
GABRG3	15	rs34984550	G	C	Intron	0.024	0.01	0.05	0.884	1.29	0.50	**9.78E-03**	0.64	0.25	**9.80E-03**
		rs12903002	T	C	Intron	0.088	0.05	0.03	**0.038**	0.04	0.12	0.738	0.02	0.06	0.681

A1 = minor allele, A2 = major allele, GABAA = gamma-aminobutyric acid type A, Beta = beta coefficients, Chr = chromosome, MAF = minor allele frequency, SE = standard error.

P values <0.05 represent the significant values and are shown in bold.
