# Supplementary material for: An association study in the Taiwan Biobank elicits the GABAA receptor genes GABRB3, GABRA5, and GABRG3 as candidate loci for sleep duration in the Taiwanese population
Source: BMC Med Genomics. 2021 Sep 16;14:223. doi: 10.1186/s12920-021-01083-x (PMC8447520; doi:10.1186/s12920-021-01083-x)
Supplement: Supplementary file 6 — Additional file 6 Table S5. Linear regression models of associations between sleep timing and six SNPs in GABAA receptor genes (e.g., GABRB3, GABRA5, and GABRG3) with evidence of an association (P < 0.05). [file 12920_2021_1083_MOESM6_ESM.pdf]

Table S5. Linear regression models of associations between sleep timing and six SNPs in GABAA receptor genes (e.g., *GABRB3* , *GABRA5*, and *GABRG3*) with evidence of an association (*P* < 0.05).

							Dominant model			Recessive model			Genotypic model		
**Gene**	**Chr**	**SNP**	**A1**	**A2**	**Region**	**MAF**	**Beta**	**SE**	**P**	**Beta**	**SE**	**P**	**Beta**	**SE**	**P**
GABRB3	15	rs79565260	A	G	Intron	0.178	-0.04	0.02	**0.016**	0.01	0.04	0.783	-0.005	0.06	0.929
		rs79465949	T	C	Intron	0.018	-0.12	0.04	**3.95E-03**	-0.11	0.39	0.772	-0.14	0.49	0.768
GABRA5	15	rs146013014	G	A	Intron	0.026	-0.02	0.04	0.578	-0.59	0.28	**0.033**	-0.74	0.35	**0.033**
GABRG3	15	rs147619342	T	C	Intron	0.017	-0.05	0.04	0.253	-0.73	0.35	**0.038**	-0.91	0.44	**0.039**
		rs1017364	G	A	Intron	0.266	0.00	0.02	0.907	-0.06	0.03	**0.044**	-0.07	0.04	0.065
		rs138047237	A	G	Intron	0.013	0.11	0.05	**0.027**	0.18	0.55	0.750	0.22	0.69	0.751

A1 = minor allele, A2 = major allele, GABAA = gamma-aminobutyric acid type A, Beta = beta coefficients, Chr = chromosome, MAF = minor allele frequency, SE = standard error.

P values <0.05 represent the significant values and are shown in bold.
