# Supplementary material for: An association study in the Taiwan Biobank elicits the GABAA receptor genes GABRB3, GABRA5, and GABRG3 as candidate loci for sleep duration in the Taiwanese population
Source: BMC Med Genomics. 2021 Sep 16;14:223. doi: 10.1186/s12920-021-01083-x (PMC8447520; doi:10.1186/s12920-021-01083-x)
Supplement: Supplementary file 7 — Additional file 7 Table S6. Linear regression models of associations between sleep duration and five SNPs in GABAA receptor genes (e.g., GABRA1 and GABRA2). [file 12920_2021_1083_MOESM7_ESM.pdf]

Table S6. Linear regression models of associations between sleep duration and five SNPs in GABAA receptor genes (e.g., *GABRA1* and *GABRA2*).

							Dominant model			Recessive model			Genotypic model		
**Gene**	**Chr**	**SNP**	**A1**	**A2**	**Region**	**MAF**	**Beta**	**SE**	**P**	**Beta**	**SE**	**P**	**Beta**	**SE**	**P**
GABRA1	5	rs142983657	T	C	Intron	0.019	0.01	0.03	0.774	0.03	0.03	0.320	-0.59	0.40	0.140
		rs2279020	A	G	Intron	0.435	-0.07	0.04	0.063	-0.11	0.25	0.654	0.02	0.02	0.326
GABRA2	4	rs28753386	C	A	Intron	0.488	0.04	0.06	0.534	-1.18	0.80	0.140	0.01	0.02	0.424
		rs76707584	A	G	Intron	0.034	-0.01	0.02	0.673	-0.03	0.04	0.396	0.01	0.17	0.969
		rs16851626	G	A	Intron	0.049	0.00	0.02	0.862	0.04	0.03	0.211	-0.06	0.13	0.634

A1 = minor allele, A2 = major allele, GABAA = gamma-aminobutyric acid type A, Beta = beta coefficients, Chr = chromosome, MAF = minor allele frequency, SE = standard error.

P values <0.05 represent the significant values and are shown in bold.
