# Supplementary material for: An association study in the Taiwan Biobank elicits the GABAA receptor genes GABRB3, GABRA5, and GABRG3 as candidate loci for sleep duration in the Taiwanese population
Source: BMC Med Genomics. 2021 Sep 16;14:223. doi: 10.1186/s12920-021-01083-x (PMC8447520; doi:10.1186/s12920-021-01083-x)
Supplement: Supplementary file 8 — Additional file 8. Table S7. Linear regression models of associations between sleep quality and two SNPs in the GABAA receptor gene GABRA2 with evidence of an association (P < 0.05). [file 12920_2021_1083_MOESM8_ESM.pdf]

Table S7. Linear regression models of associations between sleep quality and two SNPs in the GABAA receptor gene *GABRA2* with evidence of an association (*P* < 0.05).

							Dominant model			Recessive model			Genotypic model		
**Gene**	**Chr**	**SNP**	**A1**	**A2**	**Region**	**MAF**	**Beta**	**SE**	**P**	**Beta**	**SE**	**P**	**Beta**	**SE**	**P**
GABRA2	4	rs76707584	A	G	Intron	0.034	0.04	0.02	**0.035**	0.04	0.04	0.291	0.07	0.15	0.637
		rs16851626	G	A	Intron	0.049	-0.04	0.02	**0.039**	-0.01	0.03	0.564	-0.17	0.11	0.125

A1 = minor allele, A2 = major allele, GABAA = gamma-aminobutyric acid type A, Beta = beta coefficients, Chr = chromosome, MAF = minor allele frequency, SE = standard error.

P values <0.05 represent the significant values and are shown in bold.
